# Dietary fortificant iron intake is negatively associated with quality of life in patients with mildly active inflammatory bowel disease

**DOI:** 10.1186/1743-7075-10-9

**Published:** 2013-01-15

**Authors:** Jonathan J Powell, William B Cook, Carol Hutchinson, Zoe Tolkien, Mark Chatfield, Dora IA Pereira, Miranda CE Lomer

**Affiliations:** 1 MRC Human Nutrition Research, Elsie Widdowson Laboratory, Fulbourn Road, Cambridge CB1 9NL, UK; 2 Department of Gastroenterology, Guy’s and St Thomas’ NHS Foundation Trust, London, UK; 3 King’s College London, Nutritional Sciences Division, London, UK

**Keywords:** IBD, Iron intake, Iron deficiency, Quality of life, Fortificant iron

## Abstract

**Background:**

Iron deficiency anaemia and oral iron supplementation have been associated negatively with quality of life, and with adverse effects, respectively, in subjects with inflammatory bowel disease (IBD). Hence, the risk-benefit ratio of oral iron is not understood in this patient group. The present case–control study investigated whether dietary iron intake impacts on quality of life in IBD patients.

**Methods:**

Quality of life, habitual dietary iron intakes and iron requirements were assessed in 29 patients with inactive or mildly active IBD as well as in 28 healthy control subjects.

**Results:**

As expected, quality of life was worse in IBD patients as a whole in comparison to healthy controls according to EuroQol score and EuroQol VAS percentage (6.9 ± 1.6 *vs* 5.3 ± 0.6; *p*< 0.0001 and 77 ± 14% *vs* 88 ± 12%; *p*=0.004 respectively). For IBD subjects, 21/29 were iron deplete based upon serum iron responses to oral iron but, overall, were non-anaemic with mean haemoglobin of 13.3 ± 1.5 g/dL, and there was no difference in their quality of life compared to 8/29 iron replete subjects (Hb 14.0 ± 0.8 g/dL). Interestingly, total dietary iron intake was significantly *negatively* associated with quality of life in IBD patients, specifically for non-haem iron and, more specifically, for fortificant iron. Moreover, for total non-haem iron the negative association disappeared when fortificant iron values were subtracted. Finally, further sub-analysis indicated that the negative association between (fortificant) dietary iron intake and quality of life in IBD patients is driven by findings in patients with mildly active disease rather than in patients with quiescent disease.

**Conclusions:**

Iron deficiency *per se* (i.e. without concomitant anaemia) does not appear to further affect quality of life in IBD patients with inactive or mildly active disease. However, in this preliminary study, dietary iron intake, particularly fortificant iron, appears to be significantly negatively associated with quality of life in patients with mildly active disease.

## Background

Iron deficiency (ID) is common in patients with inflammatory bowel disease (IBD) (36-90% prevalence [1-3]), and when this results in moderate or severe anaemia is associated with sub-optimal quality of life [4,5]. Well-being, mood, physical activity and social activities are especially affected. Somewhat paradoxically there is concern that supplementation with oral iron, in patients with IBD, may also induce symptoms and impact quality of life [6-10] probably due to the free-radical generating activity of unabsorbed luminal iron and the detrimental effect this has on an already sensitive mucosa [11]. Indeed, in animal models of IBD, this, and the underlying mechanisms, have been demonstrated as have the knock-on sequelae in terms of colonic cancer risks including with relatively low ‘fortificant’ levels of dietary iron [12-16]. In short, therefore, the ‘risk-benefit ratio’ (i.e. balancing sub-optimal iron status with undesirable gastrointestinal effects) of lower versus higher dietary iron intakes in patients with IBD is not known. To address this issue we took advantage of a study investigating iron intakes and absorption in IBD patients [17] by additionally measuring quality of life. Unlike previous work, our cohort excluded subjects with moderate or severe anaemia. Nonetheless, such a study is not straightforward because in the absence of severe anaemia, the diagnosis of iron deficiency using routine clinical measures is flawed in subjects with IBD mainly as a result of chronic (including low grade, sub-chronic) inflammatory processes [18]. However, by measuring iron absorption in each subject we were able to separate subjects, accurately, as those requiring iron (iron deplete) and those not requiring iron (iron replete) and thus could compare these stratifications with quality of life. The pros and cons of this approach versus ‘conventional’ haematology measures are carefully addressed elsewhere [17], [Powell et al., 2012, submitted]. In addition, using an iron-specific, validated, food frequency questionnaire (FFQ) [19], we collected dietary data enabling us to investigate associations between dietary iron intakes and quality of life in IBD patients.

## Subjects and methods

### Study design

Twenty nine IBD patients (5 with ulcerative colitis and 24 with Crohn’s disease) were recruited from gastrointestinal outpatient clinics at Guy’s and St Thomas’ NHS Hospital Trust (GSTT), London, UK. Control subjects (*n*=28) were recruited from a local newspaper advert.

This study was conducted according to the guidelines laid down in the Declaration of Helsinki and all procedures involving human subjects were approved by the St Thomas’ Hospital Local Research Ethics Committee (EC03/089). Written informed consent was obtained from all subjects.

The data presented here were collected as part of a wider programme and further details including iron status, inflammatory status and absorption data have been previously published [17]. Mild anaemia was defined as a haemoglobin concentration of 11–12.9 g/dl (male) and 10.5-12.0 g/dl (female) [18]. Subjects with non-Fe-deficiency anaemia, moderate-severe disease activity (e.g. Harvey Bradshaw Index [HBI] >8 [20]) or homozygous mutations of the HFE gene were excluded from the study.

### Patients

Twenty nine patients, aged 18 to 65, were recruited from outpatient gastroenterology clinics at GSTT and in all cases IBD was diagnosed by histological and/or radiological techniques. Five patients had ulcerative colitis and 24 had Crohn’s disease. Patients with other chronic diseases, hereditary disorders of iron metabolism (detected by assessment of common mutations in the HFE gene), pregnant or lactating females and those who had received iron therapy within the previous 28 days were excluded. Additionally, only patients with inactive or mildly active disease, i.e. with an HBI in the range of 0–4 or in the range 5–8 respectively [20], were included in the study as detailed elsewhere [17]. HBI is validated for use with Crohn’s disease, as the large majority of patients had here, but, as previously [17], its use was also extended to the few ulcerative colitis patients for consistency with all patient subject measures.

### Controls

An advert was placed in a freely available newspaper distributed predominantly within Greater London. Potential subjects responding to the advert were screened by telephone to exclude anyone with known chronic disease, gastrointestinal disease, hereditary disorders of iron metabolism and those taking proton-pump inhibitor medication or iron therapy/supplements within the previous 28 days. Pregnant and lactating women were also excluded. Mutations of the HFE gene were later assessed as before [17].

### Quality of life questionnaires

Two questionnaires were administered to assess quality of life. Firstly, we used the EuroQol EQ-5D VAS, designed to measure health related quality of life across a wide range of health conditions. This was used on both the IBD subjects and the controls. Secondly, the McMaster Inflammatory Bowel Disease Questionnaire (IBDQ) was used but, by definition, only for the IBD subjects.

The EQ-5D, developed by EuroQol, is self-administered and takes less than five minutes to complete. It comprises five dimensions of health: mobility, self-care, usual activities, pain/discomfort and anxiety/depression. Each dimension has three levels: (1) no problems, (2) some/moderate problems and (3) extreme problems. Each level is allocated points: one point for no problems, two for some/moderate and three for extreme. A score is calculated by adding together the points for each dimension. The lowest possible score is five and the maximum 25. In addition subjects were asked to complete a self-rated health status on a visual analogue scale (VAS). This consisted of a vertical graduated line that the subjects used to indicate their health status on the day of the study. The bottom of the line represented the worst possible health state and the top the best imaginable health state. Subjects indicated their health status on the scale by drawing a line from a box labelled ‘Your own health state today’ to the appropriate point on the scale. Thus, two measures, namely EuroQol score and EuroQol VAS percentage were derived.

The McMaster IBDQ was the first published IBD questionnaire and has been extensively validated [21]. This questionnaire has been shown to be more sensitive in detecting change in quality of life compared with a generic questionnaire, thus indicating that it is disease specific. The questionnaire has been shown to be a reliable indicator of change in disease activity for both Crohn’s disease and ulcerative colitis [22]. The McMaster IBDQ consists of 32 questions designed to assess four different domains: (1) emotional function (2) bowel function (3) social function and (4) systemic function. Each question is scored on a seven point Likert scale. The total score value ranges from 32 to 224 points and a higher score indicates better health-related quality of life. The questionnaire was self-administered.

### Dietary iron intake

A specific FFQ was used to assess intakes of dietary iron over the previous month. The FFQ was a validated computerised quantitative food frequency questionnaire developed specifically for iron, as well as its absorption modifiers [19], although only iron, and not the absorption modifiers, was considered in this work. Fortificant iron was calculated as follows: (i) for food products which contain almost no other iron sources apart from ‘added’ iron, e.g. Fortisip drink, the iron content declared by the manufacturer was taken as fortificant iron; (ii) for food products which are fortified with iron to varying levels at the discretion of the manufacturer, e.g. breakfast cereals, the level of ‘natural’ (i.e. not added) non-haem iron was estimated based on the ingredients information using the McCance and Widdowson food composition tables [23], and this value was then subtracted from the total iron content declared by the manufacturer to estimate fortificant iron content; and (iii) for other foods containing white wheat flour, which is fortified with iron as a requirement under the UK Bread and Flour Regulation 1995, fortificant iron was estimated by considering the content of white flour per food portion and using the standard ‘restoration’ formula of 1.65 mg Fe/100 g white flour to derive the content of fortificant iron per food portion. Foods falling within this latter category, for the FFQ used in this work, are shown in Table 1. Natural non- haem iron (i.e. not-added, dietary-derived iron) was calculated by subtracting fortificant iron from total non-haem iron. All values for the different categories of dietary iron were calculated as total daily intakes for each subject.

**Table 1:** FFQ food items containing a mixture of fortificant iron from white wheat flour and iron derived from other sources (i.e. non-haem and/or haem iron)

Sponge cake	Fruit cake	Pancakes, sweet
Stuffing, sage and onion	Yorkshire pudding	Fruit pie
Quiche, cheese and egg	Croissants	Digestive biscuits
Shortbread	Lemon meringue pie	Sponge pudding
Pizza, cheese and tomato	Samosa, vegetable	Sausage rolls
Steak and kidney pie	Chicken pie	Beef pie
Iced cakes, fancy	Choux bun	Gingernut biscuits
Danish pastry	Doughnuts, jam or custard	Cream crackers
Custard tart	Coconut cake	Brown bread

### Classification of iron status

Subjects were classified as iron deplete or replete based on the serum iron curves obtained following the administration of a single 200 mg ferrous sulphate capsule (equivalent to 65 mg elemental Fe). Methods and results of this comparative absorption study have been recently published [17]. Iron deplete were defined as having a rise in serum iron greater than 5 μmol/L from baseline in a four-hour period following ingestion of 65 mg of iron as ferrous sulphate [17]. Iron replete had a serum iron rise of less than 5 μmol/L over the same period of time [17]. This classification was used to predict iron requirements as traditional clinical haematological parameters (ferritin, transferrin saturation etc.) are not a reliable indicator of iron status in IBD patients [17,18] and, apart from bone marrow staining, iron absorption is the best measure of iron requirements [21,24].

### Statistical analysis

Comparisons between healthy controls and IBD patients were made using unpaired *t* tests. Significance was assumed at *p* ≤ 0.05. Values expressed are mean ± standard deviation. Significance of associations between the different fractions of dietary iron intake and quality of life were assessed using simple linear regression (Pearson’s product moment correlation coefficient ) and multiple linear regression (where appropriate).

## Results

As expected, EuroQol score and EuroQol VAS percentage were significantly different between IBD patients and healthy control subjects (6.9 ± 1.6 *vs* 5.3 ± 0.6; *p*< 0.0001 and 77 ± 14% *vs* 88 ± 12%; *p*=0.004).

Next, we compared quality of life scores between IBD patients that were iron deplete and iron replete. Twenty one patients were classified as iron deplete (4/21 having concomitant mild anaemia) and eight as iron replete (none with concomitant anaemia). Respectively, there were no significant differences in quality of life measured using EuroQol score (7.0 ± 1.8 *vs* 6.6 ± 0.8; *p*=0.46), EuroQol VAS percentage (79 ± 14% *vs* 75 ± 17%; *p*=0.56) or McMaster IBDQ score (157 ± 36 *vs* 155 ± 15; *p*=0.84).

**Figure 1:**
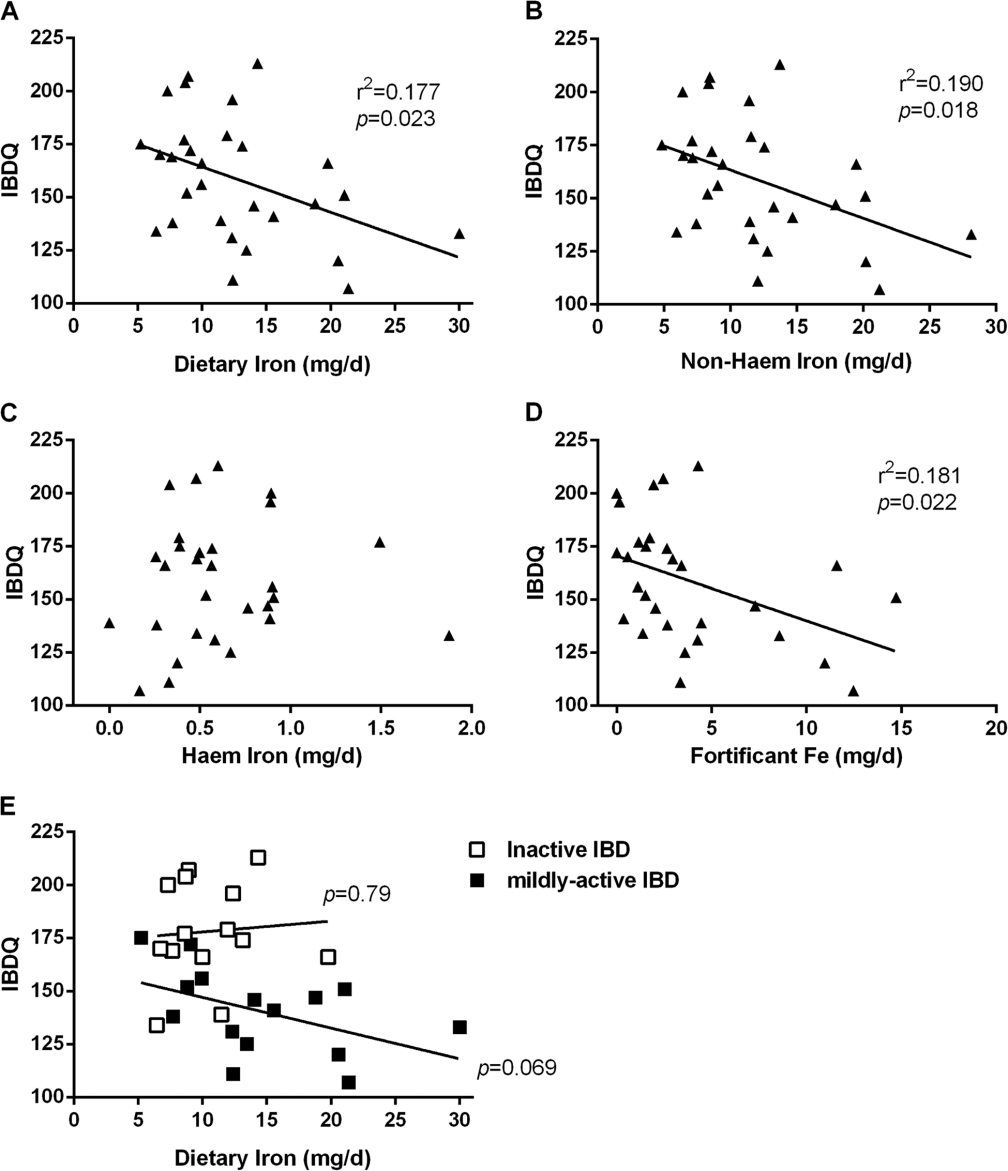
**Association between quality of life measured with McMaster IBDQ with A) total dietary iron, B) non-haem iron, C) haem iron, and D) fortificant iron in IBD patients: E) association between IBDQ score and dietary iron intake as per a) in relation to disease activity. **r, Pearson correlation coefficient; *p*, p-value for the slope deviation from zero.

**Figure 2:**
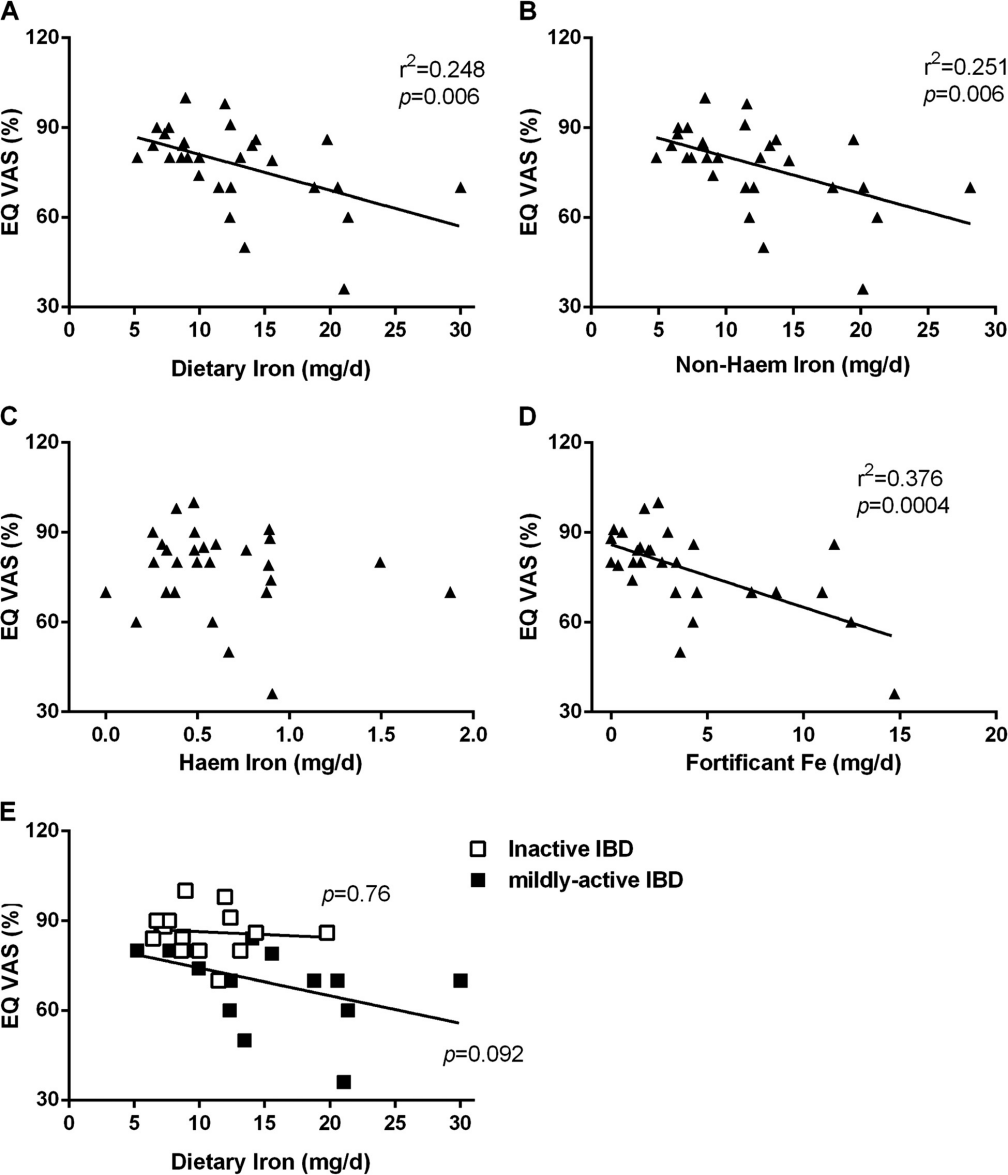
**Association between quality of life measured with EuroQol VAS percentage with A) total dietary iron, B) non-haem iron, C) haem iron and D) fortificant iron in IBD patients: E) association between EuroQol VAS percentage and dietary iron intake as per a) in relation to disease activity. **r, Pearson correlation coefficient; *p*, p-value for the slope deviation from zero.

**Figure 3:**
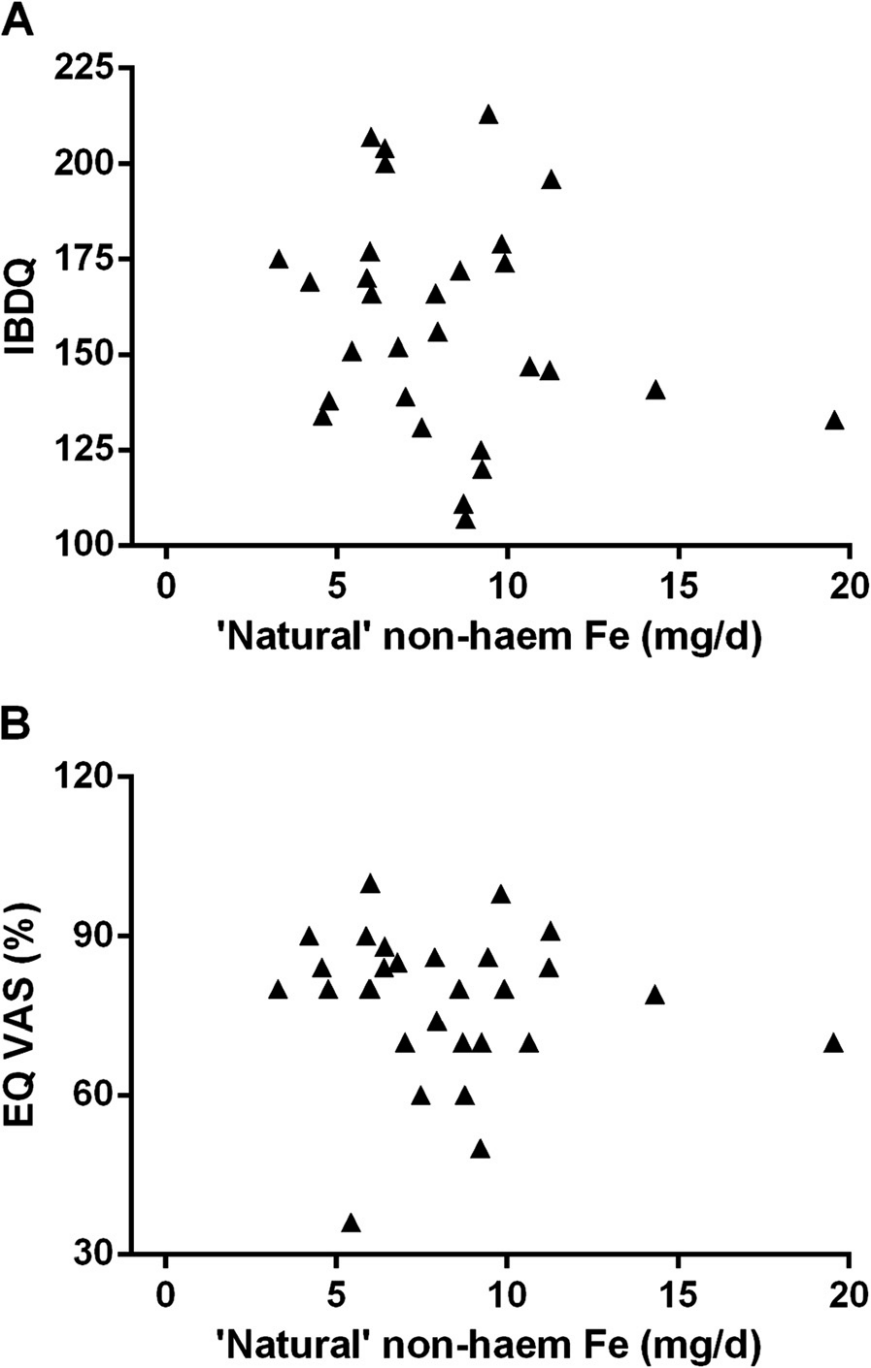
**Association between quality of life measured with A) McMaster IBDQ and B) EuroQol VAS percentage for ‘natural’ dietary non-haem iron in IBD patients. **Natural non-haem iron represents not-added, dietary-derived iron, calculated by subtracting fortificant iron from total non-haem iron.

Interestingly, when looking at the association between total dietary iron, non-haem iron, haem iron and fortificant iron intakes, against scores for quality of life in IBD patients as measured by the McMaster IBDQ (Figure 1) or the EuroQol VAS percentage (Figure 2), significant negative associations were observed for total dietary iron, non-haem iron and fortificant iron. Moreover, once the fortificant iron values were removed (i.e. subtracted) from non-haem iron intakes, to leave only ‘natural’ (naturally dietary-derived) non-haem iron, the significant negative association disappeared (Figure 3). Moreover, there remained a significant negative association between fortificant iron and quality of life measured by either IBDQ score (*p*=0.041) or EuroQol VAS percentage (*p*=0.005) when using multiple linear regression and considering fortificant iron, haem iron and ‘natural’ non-haem iron as independent variables (predictors) and quality of life as the dependent variable.

Finally, in dividing the IBD patient group into those with quiescent disease (i.e. HBI 0–4) and those with mildly active disease (i.e. HBI 5–8) it was clear that the above associations were driven by findings in patients with (mildly) active disease rather than in quiescence, although the small subgroup numbers do not enable robust statistical analysis (Figures 1E and 2E).

No associations were observed between the EuroQol score and iron intakes (not shown), nor for control subjects with any quality of life measure and iron intakes (not shown).

## Discussion

Studies have shown that increasing haemoglobin levels, for those with moderate or severe anaemia, improves quality of life in IBD patients [4,25,26] and therefore one might expect iron status to reflect quality of life. However, results from this small study show that, in the absence of moderate or severe anaemia, there is no significant difference in quality of life between IBD patients classified as iron deplete and iron replete. As noted previously, categorisation as iron replete/deplete is superior when based upon oral iron challenge (as here) rather than standard haematological parameters that can be disturbed in this patient group [17,18,27]. The lack of observed difference could be explained by the fact that, in this work, only 4/21 of the iron deplete patients had anaemia (which was anyway mild) and, overall, the iron deplete group had a mean haemoglobin level of 13.3 g/dL [17] (i.e. within the normal reference range even if iron stores where low). Similar work in patients with cancer showed the biggest improvement in quality of life when haemoglobin levels increased from 11 to 13 g/dL [28]. Therefore, the iron deplete patients in our study appear, in the main, have haemoglobin levels above the threshold for the detection of deterioration in quality of life. From this we can conclude that iron deficiency in the absence of anaemia does not have a measurable effect on quality of life in IBD. This is important information because there is evidence that oral iron supplementation can have detrimental effects on the symptoms of IBD [5,6,9,10,29,30], so only supplementing when necessary seems prudent.

Moreover, for the IBD patients reported herein, we found that their iron intakes, and in particular their intakes of fortificant iron, were significantly *negatively* associated with quality of life, as measured by both the McMaster IBDQ and EuroQol VAS percentage. However, this association appears to ‘show up’ only in patients with some degree of disease activity (Figures 1E and 2E), although we accept that patient numbers were low in these subgroups and a larger study would be required to see if there really is no such association in patients with quiescent disease.

Animal models have indicated that relatively small changes to luminal iron in the colon can lead to exacerbation of local inflammatory effects (and their biomarkers) [12,13,15] and, similarly, that removal of luminal iron can alleviate local inflammation [16]. However, the gastrointestinal effects of dietary/fortificant iron in humans with IBD is not understood [31] and, so, these are the first data to suggest that what is observed in animal models of IBD may also be observed in the population with non-quiescent IBD. Nevertheless, we recognise that the numbers studied here were small (n=29 overall and n=15 with mildly active disease) and that the less sensitive measure for quality of life in these subjects (i.e. the generic EuroQol score) did not detect any association. Hence, the study really needs repeating with a larger cohort, preferably longitudinally, but, if other findings are consistent, then this would add further credibility to work suggesting that unabsorbed dietary iron can have adverse effects in the colon and especially in those with pre-existing inflammation.

If the above findings were verified, how might dietary fortificant iron be mediating its effect in sensitive individuals? After all, compared to oral iron supplementation (typically 60–120 mg Fe/day) the amount of iron in the diet, even allowing for fortification, is low (5–25 mgFe/day in this study) and whilst iron supplementation may be problematic in some subjects with IBD [5,6,9,10,29,30] it is clearly not to the extent that would be expected if dietary iron alone were a problem. This is a puzzle that deserves consideration although it is possible that there is a low dose threshold for the iron effect or that long term iron-effects are required (e.g. changes in colonic flora [16]) that are not irreversibly experienced with the shorter duration of supplementation. Finally, the chemical form of the iron should be considered: for example, it is worth noting that an inflammatory murine model of ulcerative colitis showed that a mere doubling of dietary iron, using iron EDTA, led to a 4–5 fold increase in tumour incidence [32]. Even in the small study presented here, the range of iron intakes was about four-fold.

To our knowledge the study presented herein is the first study carried out in humans to investigate the impact of dietary iron, particularly from iron-fortified products, on quality of life. We focused, especially, on IBD patients as a potentially vulnerable and sensitive (to effects) population. This study can be viewed as hypothesis-generating and we propose that further studies are undertaken to determine whether high dietary iron intakes are, reproducibly, negatively associated with quality of life in IBD patients (with or without active disease) and, if so, firstly if this is related to endogenous food-iron or fortificant iron (for the non-haem fraction) and, secondly, if the issue extends to the wider population in terms of dietary iron and long-term distal gut health. In which case, for a healthy population without pre-existing gut mucosal sensitivity, it is unlikely that symptoms (quality of life) would be a sufficient indicator and biochemical, microbial or histological parameters would be required.

## Abbreviations

IBD: Inflammatory bowel disease; ID: Iron deficiency; IDA: Iron deficiency anaemia; GSTT: Guy’s and St Thomas’ NHS Foundation Trust; FFQ: Food frequency questionnaire; VAS: Visual analogue scale.

## Competing interests

The authors do not have financial conflicts of interest of any kind, nor have personal relationships with other people or institutions that could inappropriately influence our work.

## Authors’ contributions

The authors’ responsibilities were as follows: MCEL and JJP designed the study. MCEL and WBC carried out the study. WBC, CH, ZT, MC and DIAP carried out data analysis. All authors have contributed to the preparation of the manuscript and have approved the manuscript. JJP and DIAP had primary responsibility for the final content of the manuscript.
